# The vestibular implant: effects of stimulation parameters on the electrically-evoked vestibulo-ocular reflex

**DOI:** 10.3389/fneur.2024.1483067

**Published:** 2024-11-06

**Authors:** Stan C. J. van Boxel, Bernd L. Vermorken, Benjamin Volpe, Nils Guinand, Angélica Perez-Fornos, Elke M. J. Devocht, Raymond van de Berg

**Affiliations:** ^1^Division of Vestibular Disorders, Department of Otorhinolaryngology and Head and Neck Surgery, Maastricht University Medical Center, Maastricht, Netherlands; ^2^Mental Health and Neuroscience Research Institute (MHeNs), Maastricht University, Maastricht, Netherlands; ^3^Service of Otorhinolaryngology Head and Neck Surgery, Department of Clinical Neurosciences, Geneva University Hospitals, Geneva, Switzerland

**Keywords:** vestibular implant, vestibular stimulation, electrically evoked vestibulo-ocular reflex, stimulation amplitude, phase duration, pulse rate

## Abstract

**Introduction:**

The vestibular implant is a neuroprosthesis which offers a potential treatment approach for patients suffering from vestibulopathy. Investigating the influence of electrical stimulation parameters is essential to improve the vestibular implant response. Optimization of the response focuses on the electrically evoked vestibulo-ocular reflex. It aims to facilitate high peak eye velocities and adequate alignment of the eye movement responses. In this study, the basic stimulation parameters of the vestibular implant were tested for their effect on the electrically evoked vestibulo-ocular reflex.

**Methods:**

Four stimulation parameters, including the stimulation amplitude, phase duration, stimulus rate and speed of change of stimulation, were systematically tested in a cohort of nine subjects with a vestibulo-cochlear implant. These parameters were tested to evaluate their effect on fitting settings (i.e., threshold of activation, upper comfortable limit and dynamic range) as well as on the electrically evoked vestibulo-ocular reflex (peak eye velocity and alignment).

**Results:**

It was confirmed that, in addition to current amplitude, the peak eye velocity of the response can be increased by increasing the phase duration and pulse rate. Both parameters have little effect on the alignment of the eye response. However, a longer phase duration decreased the range between the threshold of activation and the upper comfortable limit of the electrical stimulation (i.e., dynamic range). Furthermore, these results show that next to the amplitude of the stimulation, the speed of change in stimulation has a determinative positive effect on the peak eye velocity.

**Conclusion:**

The observations in this study imply that the vestibular implant response, in terms of peak eye velocity, can be optimized with a higher pulse rate and longer phase duration. However, this comes at a trade-off between the dynamic range and power consumption. This study provides essential insights for fitting strategies in future vestibular implant care.

## 1 Introduction

The vestibular system is crucial for essential tasks such as postural control, gaze stabilization and spatial orientation. The vestibular end organ, located in the inner ear, detects head movements using three semicircular canals (sensitive to rotation in three orthogonal planes), and two otolith organs (sensitive to translations and gravity). This information is used to, e.g., facilitate the vestibulo-ocular reflex (VOR) to establish gaze stabilization, and the vestibulo-collic and vestibulo-spinal reflexes to maintain postural control (1). Impairment of the vestibular system leads to a diminished ability to perform those tasks, resulting in a reduced quality of life (2). Although the prevalence of vestibulopathy is estimated to be considerable, between 53 and 95 million in Europe and the United States (3), effective treatment to restore vestibular function remains unavailable (4, 5).

As a novel treatment approach, multiple research groups are investigating the potential of a vestibular implant. This is a neuroprosthesis implanted in the inner ear, analogous to a cochlear implant (6). A vestibular implant provides movement information to the brain, by electrically stimulating the nerve ends in the vestibular organs, therefore bypassing the defective sensors. It was demonstrated that a vestibular implant can (partially) restore vestibular function (7–11).

**Figure 1:**
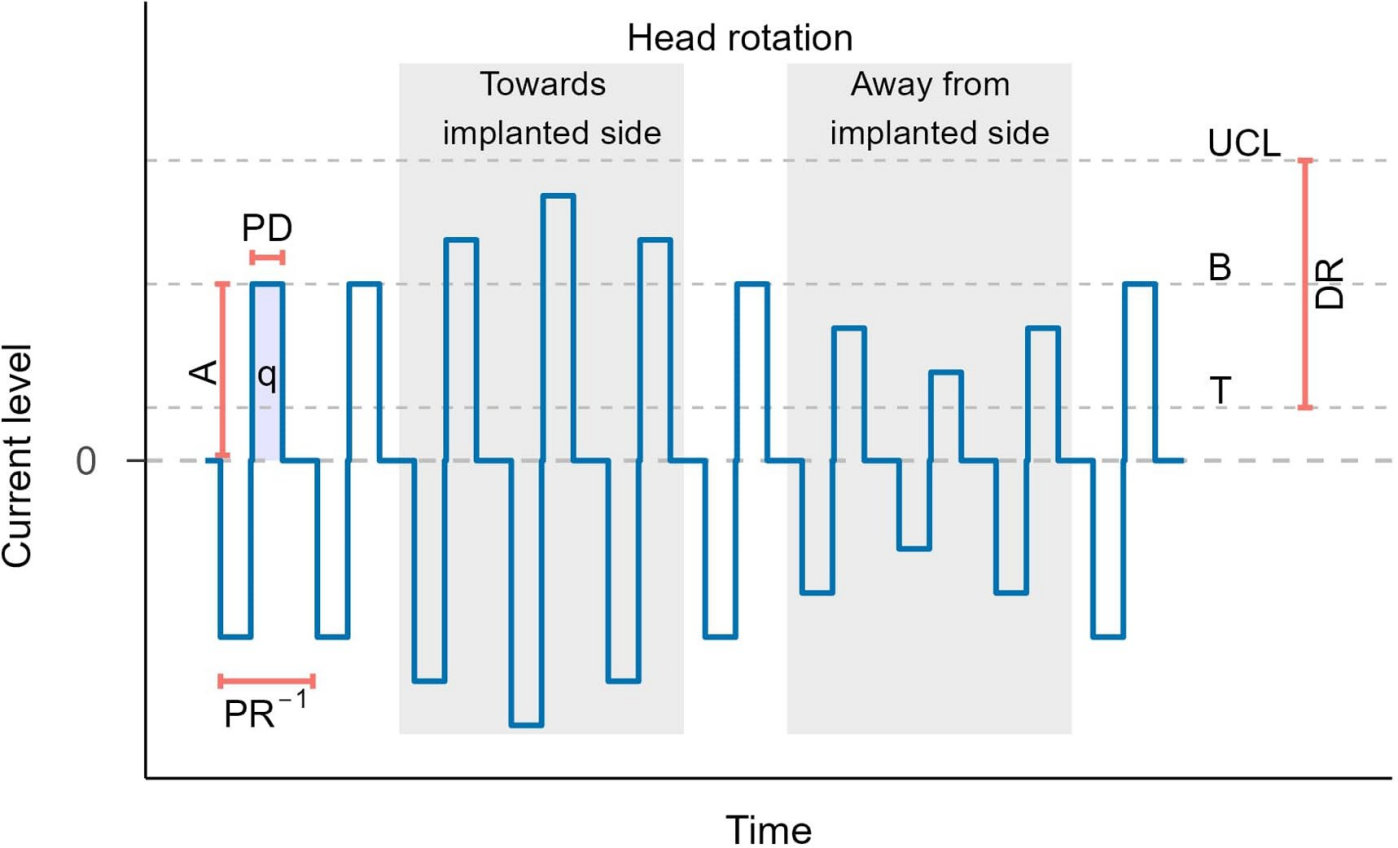
Schematic visualization of the stimulation pattern used in vestibular stimulation for horizontal head movements. A, amplitude; PD, phase duration; q, charge; PR^−1^, pulse rate^−1^; UCL, upper comfortable limit; B, baseline; T, threshold; DR, dynamic range. For better visualization, the phase duration is displayed longer than it actually is relative to the stimulation rate.

In the healthy vestibular system, the vestibular afferents in the semicircular canals and otolith organs fire at a constant baseline rate. As a result of head rotations, the firing rate of the afferents in the semicircular canals increases or decreases depending on the direction in which the head moves. The magnitude of change in rate depends on the dynamic properties (e.g., velocity) of the rotations. Electrical vestibular stimulation with a vestibular implant can be used to mimic this mechanism. Currently, this comprises a baseline stimulation of charge balanced, cathodic first, biphasic, rectangular pulses, with a specific rate and amplitude level (see schematic visualization in Figure 1) (9, 12). For the lateral canal, this stimulation is increased (amplitude and/or rate) to encode head rotations towards the implanted side, and decreased for the opposite direction. For the superior and posterior canals, stimulation is increased when the rotation has a (approximately) forward and backward orientation, respectively. By stimulating the three semicircular canals separately, vestibular information can be provided in all three planes of space.

A goal of vestibular stimulation is to evoke compensatory eye movements, namely the electrically evoked vestibular-ocular reflex (eVOR), that reestablish gaze stabilization. Therefore, vestibular implant stimulation should be capable of evoking eye responses with a high peak eye velocity (PEV). This facilitates encoding of a wide range of head rotations (8, 13, 14). In addition, the eVOR response should preferably be aligned with the target canal. This means that, for example, when stimulating the horizontal canal, a purely horizontal eye movement should be elicited. The activation of adjacent canal neurons should be limited to reduce the possibility of deviated alignment of the eye movements response (5, 15, 16). Furthermore, stimulation of other nearby structures, such as the facial nerve, should be avoided as well. Lastly, it is hypothesized that analogous to a cochlear implant, the functional range of electrical stimulation, also known as the electrical dynamic range (DR), should be sufficient. This is defined as the range between the threshold level (T), being the lowest current level to elicit an effect (eVOR or perception), and the upper comfortable limit (UCL), being the highest current level below undesired effects (e.g., patient discomfort or facial nerve stimulation). Because information about head movements is provided by differences in stimulation, these differences should be noticeable by the central nervous system. A larger dynamic range enables larger stimulation differences, which potentially facilitate a clearer interpretation by the central nervous system. However, this is still a hypothesis, and no minimally required dynamic range values are available. Furthermore, next to undesired effects, the maximum stimulation level can also be limited by the compliance limit of the implant (i.e., the maximum stimulation output). This makes the compliance limit an additional factor to consider when optimizing stimulation parameters to maximize the dynamic range.

To optimize PEV, alignment and dynamic range, it is key to investigate the effect of multiple stimulation parameters on those response characteristics. These stimulation parameters include the current amplitude, phase duration, pulse rate and the stimulus shape, as visualized in Figure 1. These parameters are directly proportional to the delivered cumulative charge of a pulse train (demonstrated in Figure 1 as the area under the curve). The eye movement response in turn is also proportional to the delivered cumulative charge. However, it is not yet clear whether the same amount of delivered cumulative charge obtained using different combinations of stimulation parameters (e.g., low amplitude and long phase versus high amplitude and shorter phase) will result in equivalent responses (15).

A first key parameter to control vestibular responses is the current amplitude, with higher amplitudes evoking eye responses with higher peak eye velocities (7). However, higher amplitudes might result in larger spread of excitation, with the risk of exciting non-target neurons. This could lead to misalignment of the eye movement response (17).

Secondly, the phase duration is known to be a relevant stimulation parameter (15, 16). Considering pulses with equal current amplitudes, a longer phase duration results in more delivered charge, with potentially a higher response. This can be valuable for increasing PEV, while staying within compliance limits. However, stimuli with shorter phase durations need less charge to reach nerve activation and result in less spread of excitation (15, 16, 18–21). By reducing spread of excitation, a shorter phase duration might reduce the degree of misalignment and increase the threshold for facial nerve stimulation. An increased facial nerve stimulation threshold could lead to an increased UCL, and thus a larger dynamic range (Figure 1) (16). However, this does not necessarily lead to an increase in PEV (16). Additionally, a shorter phase duration enables a higher stimulation rate (see next paragraph). This combination is more effective than a longer phase duration with a lower stimulation rate (22, 23). As a consequence, a shorter phase duration with a higher stimulation rate and should reduce power consumption.

Thirdly, the stimulation rate is expected to be a factor of influence in vestibular implant fitting. A higher stimulation rate enables a higher temporal resolution of the transferred information. In other words, information about changes of head movements over time can be more precisely provided by the implant. Furthermore, to a certain extent, a higher stimulation rate is capable of evoking stronger responses (15, 16, 24–26). This could be explained by the fact that the healthy vestibular system is based on firing rate of the sensory epithelium (1). Furthermore, a higher stimulation rate results in more delivered charge, which to a certain extent, also leads to the activation of more neurons (24, 26).

Lastly, next to the amplitude, phase duration and stimulation rate, the stimulus shape (e.g., square, trapezoid or sinusoidal) could also influence the response to stimulation. The stimulus shape influences the speed of change in stimulation, e.g., transitioning instantaneously from zero to maximum in a square pulse, or gradually following a slope in a sinusoidal pulse. Testing this influence will further clarify whether the response is dominated by the amplitude and/or delivered charge, or by the speed of change in electrical stimulation. This can be evaluated by comparing responses evoked by sinusoidal (relatively slow change in stimulation) and block (fast change) stimulations. Previous research showed that frequency dependency of the vestibular implant response is equivalent to the healthy vestibular system, with greater eye response at higher frequencies (27). This implies that the system is more sensitive to changes in stimulation than to absolute stimulation levels (in terms of amplitude and delivered charge). Those effects, together with potential effects of the stimulus shape on activation threshold, UCL and dynamic range, are important to consider when developing future vestibular implant fitting strategies.

As mentioned before, a key topic in the development of a clinically applicable vestibular implant is the encoding of vestibular information into electrical stimulation, which should evoke adequate eye and postural responses. Therefore, the objective of this study was to systematically test the effect of different stimulation parameters on the electrically evoked vestibulo-ocular reflex, using an investigational vestibulo-cochlear implant in a cohort of nine subjects with bilateral vestibulopathy. The effect of current amplitude, phase duration, stimulation rate (and related charge) and shape of stimulation pulses on the vestibulo-cochlear implant response was studied. The response was evaluated based on threshold, UCL, dynamic range, PEV and alignment. Determining the influence of these parameters could optimize vestibular implant fitting.

## 2 Methods

### 2.1 Subjects, implant, and surgery

This study was conducted as part of the VertiGo!-trial (ClinicalTrials.gov Identifier: NCT04918745). Subject inclusion, surgery and implant were described in detail by Vermorken et al. (28). In short, nine patients with bilateral vestibulopathy with severe sensorineural hearing loss in at least the ear to be implanted, received an investigational vestibulo-cochlear implant (supplied by MED-EL, Innsbruck, Austria). Vestibular electrode implantation was performed using the intralabyrinthine approach. Real-time fluoroscopy-guidance and pre-and intraoperative 3D imaging (CT and MRI) were used to optimize and verify electrode placement within 1 mm of the ampulla (29). Subject characteristics are summarized in Table 1. The implant electrode consisted of three vestibular electrode leads, inserted in the ampulla of each semicircular canal, and an electrode array inserted in the cochlea. Vestibular target nerves were the lateral ampullary nerve (LAN), superior ampullary nerve (SAN) and posterior ampullary nerve (PAN). The implant was controlled by dedicated research software (AmpFit, MED-EL, Innsbruck, Austria). This software provided stimulation signals via a research audio-motion processor, to a radiofrequency coil secured on the implant.

**Table 1:** Subject characteristics.

Subject ID	Sex	Age at implantation (years)	Etiology bilateral vestibulopathy	Duration BV symptoms (years)	Surgery year	Implant side
VCI-1	Female	54	DFNA-9	7	2021	R
VCI-2	Male	65	Auto-immune (CREST)	21	2021	R
VCI-3	Male	52	DFNA-9	30	2022	L
VCI-4	Male	66	DFNA-9	10	2022	R
VCI-5	Male	28	Idiopathic	4	2022	R
VCI-6	Male	66	M. Meniere	25	2022	R
VCI-7	Female	62	DFNA-9	6	2022	L
VCI-8	Male	63	Skull base fracture	<1	2023	R
VCI-9	Female	62	Skull base fracture	<1	2023	R

### 2.2 Experimental design

Each subject participated in a testing period of four consecutive days during which the vestibular implant stimulation parameters of interest were systematically tested. In each experiment, a stimulus was given on a single electrode (i.e., one semicircular canal) during which the eye movements were tracked using video-oculography. Directly after the stimulus, the patient’s perception was collected. A visual analog scale (VAS) was used to define the perceived intensity of the response. A 0–10 scale was used, with zero indicating no perception, and 10 being too strong.

The influence of the stimulation amplitude, phase duration and stimulation rate was evaluated by applying block stimulations of 2-second pulse trains. Pulse trains consisted of symmetric, biphasic, rectangular, cathodic first pulses, with an interphase gap of 2.1 μs. The amplitude, phase duration and pulse rate were varied, depending on the experiment.

**Figure 2:**
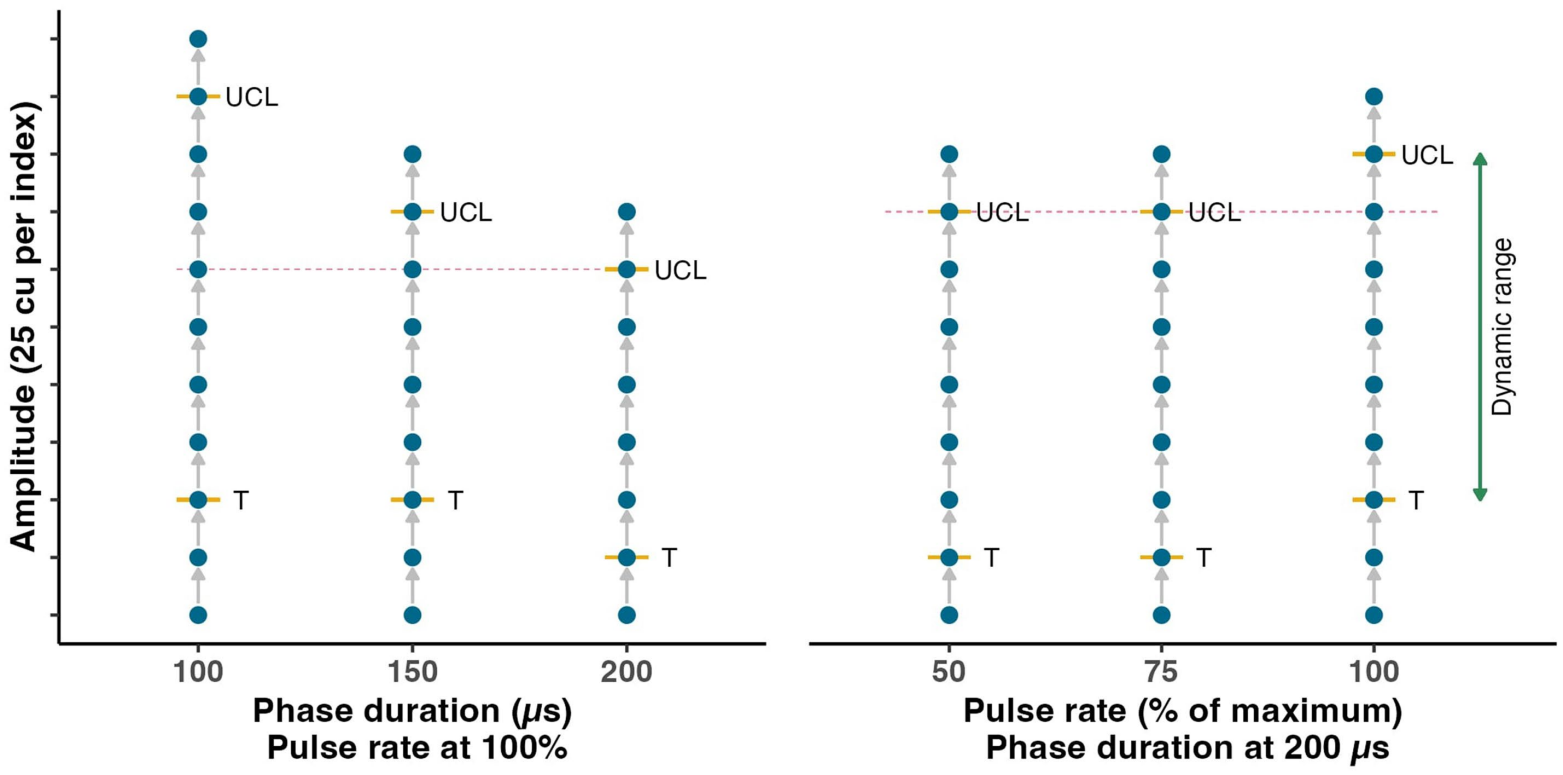
Schematic representation of the experimental procedure to evaluate the influence of the stimulus amplitude, phase duration and pulse rate. The dynamic range was determined six times for each electrode with different phase duration and pulse rate settings (i.e., each vertical line). Stimulation amplitude was increased stepwise (visualized by blue dots) until threshold of activation (T) was reached. Subsequently, the amplitude was further increased until the upper comfortable limit (UCL) was found. At each step, eye movement responses were measured and the patient’s perception was collected. The comparison among different settings with equal amplitude was made at the highest level measured in each group of three measurements (indicated by the dotted magenta colored line).

For each tested condition (i.e., combination of phase duration, stimulus rate and pulse shape), the dynamic range was determined by iteratively increasing the amplitude of the stimulation. A schematic representation of the conducted experiments is displayed in Figure 2. Stimulation started at a low level, was then increased [with steps of 50 current units (cu, with 1 cu ~ 1 mA)] to threshold (defined as the lowest current level with a vestibular perception and/or VOR), and further increased until UCL was reached (defined as the highest level without facial nerve stimulation and a VAS below 10). The threshold and UCL were determined with an accuracy of 25 cu, using a two-up-one-down-staircase procedure, checking T and UCL twice.

### 2.3 Stimulation settings

The influence of the phase duration was evaluated at 100, 150, and 200 μs. For the phase duration experiment, the stimulation rate was fixed at the maximum stimulation rate achievable by the implant. The maximum rate was patient specific as it depended on the cochlear implant fitting (inherent to the design of the used audio-motion processor) and approximated 340 pulses per second (range 322–388 pulses per second). The stimulation rate was tested for 100, 75 and 50% of the maximum achievable rate, with a fixed phase duration of 200 μs.

The effect of the phase duration and stimulus rate on PEV and alignment was evaluated at a fixed stimulation amplitude, for both factors independently. It was expected that the potential response differences between phase durations and pulse rates were most pronounced at higher stimulation levels. Therefore, the comparison was made at the highest amplitude level that was measured at all three phase duration/stimulation rate levels for each individual electrode (see vertical line in Figure 2). Due to potential differences in UCL when applying different phase durations or stimulation rates, the tested range of amplitudes could differ.

The amplitude, phase duration and stimulation rate all directly change the delivered cumulative charge of the stimulation. The charge per second is defined by q=amplitude∗phaseduration∗stimulationrate . Doubling one of the parameters results in doubling the delivered charge. The charge comparison was made by evaluating activation thresholds, UCL and PEV differences.

Differences in threshold and UCL were calculated as the differences between three levels for the phase duration (100, 150, and 200 μs) and pulse rate (100, 75 and 50%). The relative PEV increase comparison was made pairwise based on two experiments. The charge was doubled by doubling the amplitude or phase duration, and by doubling the amplitude or stimulation rate. The measurements with the doubled charge had the same settings within the comparison (i.e., amplitude, phase duration and rate). The relative PEV change was calculated by dividing the doubled charge PEV by the initial PEV.

The effect of the stimulus shape was tested by conducting experiments with block (2 s) and sinusoidal (2 Hz, 10 s) stimulations. Stimulations were tested with the phase duration fixed at 200 μs and the stimulation rate at the maximum achievable level by the implant (see above). The block/sinusoid comparison was made by modulating a continuous baseline stimulation at a current level of 50% of the dynamic range. Amplitudes were increased by steps of 10% of the dynamic range until the UCL was found, which could deviate from the UCL without baseline. Equivalent charge PEV comparison was made by comparing sinusoidal stimulation at 100% of the dynamic range versus block stimulation with an amplitude of 85%. The experiment with the sinusoids was not conducted in subject VCI-1, as this component was added later in the trial.

### 2.4 Eye tracking

During stimulation, eye movements were tracked using the VisualEyes™ goggle system (Interacoustics, Middelfart, Denmark). The goggles subjected the subject to complete darkness, and therefore prevented visual fixation. Eye responses were analyzed using the Kingslab software (Maastricht University, Maastricht, the Netherlands), and custom eye movement analysis software (supplied for the study by MED-EL, Innsbruck, Austria). Peak eye velocity values were calculated based on the maximum velocity in the direction of the eye movements. For block stimulations, the PEV of the fastest nystagmus beat was used (slow phase). Responses to sinusoidal stimulations were analyzed by calculating the PEV of half sinus fits to filtered eye response traces (30). Only artifact-free cycles were included in the analysis. Misalignment of the response was calculated as the angle between the eye response and the horizontal plane for the lateral canal, and the vertical plane for the posterior and anterior canal. Although a torsional component could be present in the eye movement responses, this was not taken into account as the used eye tracker was not capable of measuring torsion.

### 2.5 Statistics

All statistical analyses were performed using R Statistical Software (v4.3.1; R Core Team 2023). As subjects and electrodes showed substantial differences, results were normalized to facilitate the comparison of eye movement responses. Normalization was performed within each electrode measurement series. Except for misalignment, normalization involved dividing by the patient and electrode specific reference value. For example, in the comparison of the phase duration for subject VCI-1 using the LAN-electrode, the resulting PEV values were normalized by dividing by the value obtained at a phase duration of 100 μs. In contrast, misalignment was normalized by subtracting, instead of dividing by, the specific reference value (as in the previous example, the misalignment at a phase duration of 100 μs).

Means and standard deviations of the normalized values were used to quantify the effect of the stimulation parameters. Correlation coefficients were used to investigate whether the effect of the stimulation parameter was related to the level at the reference value. For example, whether the effect of the parameters were stronger for subjects showing high PEV responses. The correlation coefficient was determined between the PEV at the reference value (for the phase duration at 100 μs; for the pulse rate at 50%) and the PEV difference between the reference value and the two other settings (for the phase duration 150 and 200 μs; for the pulse rate 75 and 100%).

The inter-test variability of the PEV of the response was found to be high for PEV values below 30°/s. Therefore, the analyses were run with all samples, as well as only samples with a PEV greater than 30°/s. General conclusions were similar in both comparisons. However, to improve the reliability of the resulting effect measures, a cut off of 30°/s was used for the PEV and misalignment analyses reported here. No cut-off was applied when visualizing the patient specific amplitude to PEV response function. A cut-off of 10°/s was used for the visualization of the amplitude misalignment comparison.

In general, descriptive statistics were used. The sample size was too limited for the required multilevel tests for statistical testing. Only the charge comparison was tested for statistical significance. The distribution of the data was tested for normality using the Shapiro–Wilk test (cut-off *p* > 0.05). In case of proven normality, the paired samples two-sided t-test was used, otherwise, the paired samples Wilcoxon signed-rank test was used (both cut-off of clinical significance was *p* < 0.05). When interpreting the results over all subjects and electrodes, it should be noted that the data was nested within subjects and over electrodes.

### 2.6 Ethical considerations

This protocol (VertiGo!-trial, ClinicalTrials.gov Identifier: NCT04918745) was approved by and carried out in accordance with the recommendations of the local ethics committee (Maastricht University Medical Center, NL73492.068.20/METC 20–087). The study was designed in accordance with the declaration of Helsinki. Subjects provided written informed consent and received compensation of their travel and accommodation costs.

## 3 Results

Eight out of nine subjects, showed eVOR responses as a result of electrical stimulation of all three ampullary nerves (total of 24 electrodes). In all nine subjects, stimulation was possible within a dynamic range (>50 cu), all well within compliance limits of the device. VCI-03 did not exhibit an eVOR response on any electrode, therefore this patient was excluded from PEV and misalignment analyses. However, this patient did have a dynamic range based on perception, and was therefore included in threshold, UCL and dynamic range analyses.

### 3.1 Effect of stimulation amplitude

**Figure 3:**
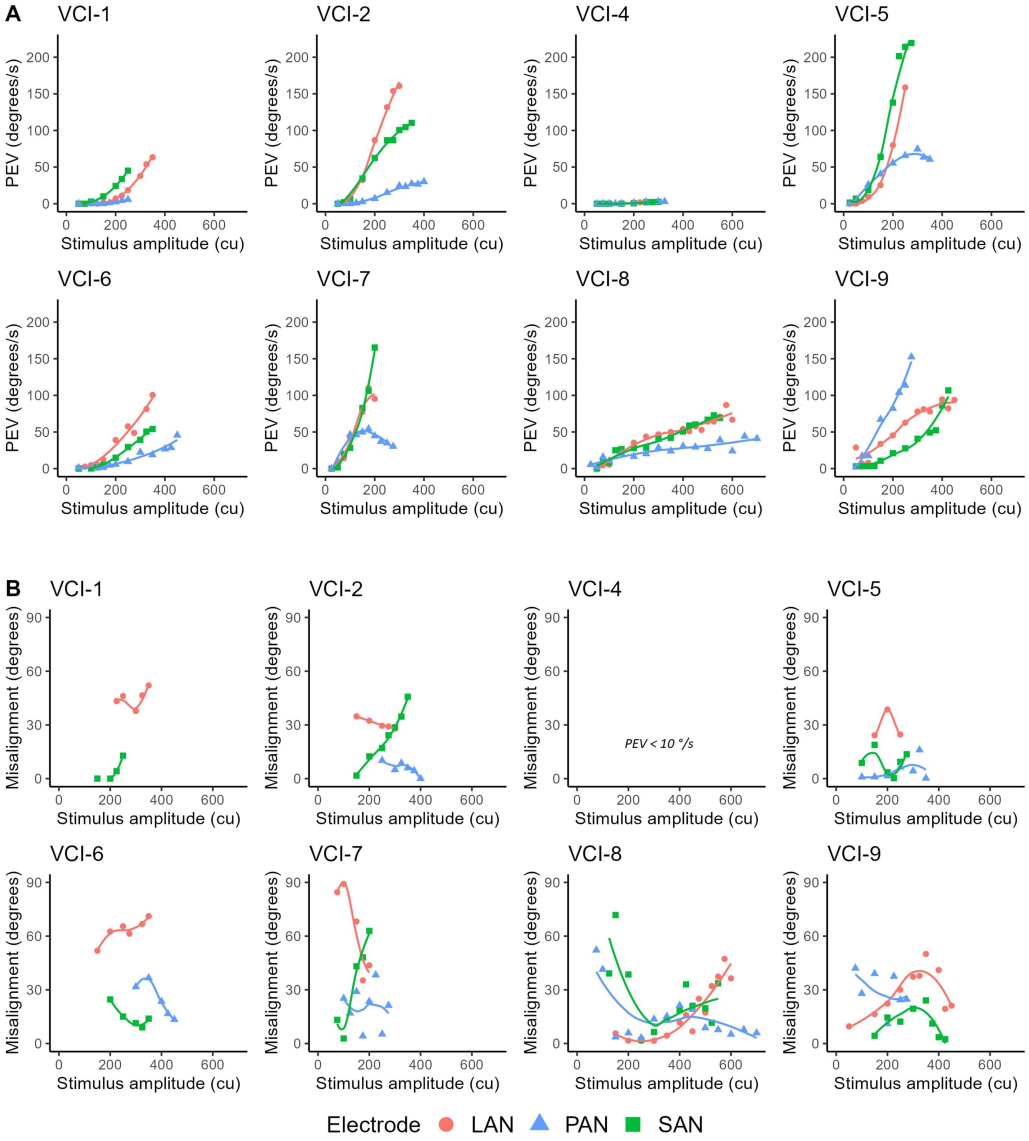
**(A)** Peak eye velocities of eVOR responses (PEV) as a function of stimulus amplitude (in current units = cu) per subject and for all three electrodes (LAN, lateral ampullary nerve; SAN, superior ampullary nerve; PAN, posterior ampullary nerve). Markers were connected using a spline fit. VCI-3 did not show eVOR responses and is therefore not shown. Stimulations consisted of 2 s block stimulations at a phase duration of 200 μs and maximum pulse rate (approximately 340 pulses per second). **(B)** Misalignment for all responses with a PEV >10 °/s (VCI-1 SAN and VCI-4 LAN/SAN/PAN not shown).

Figure 3A demonstrates the effect of the current amplitude on the PEV. Maximum PEV ranged from a few degrees per second (e.g., VCI-4) to over 200 °/s (VCI-5). In general the PEV steadily increased with increasing amplitude. At certain amplitude levels in several subject’s electrodes (VCI-4, VCI-5 and VCI-7) the PEV no longer increased, or even decreased when the amplitude was further increased. In general, the PEV at LAN and SAN stimulation was higher than at PAN stimulation (except VCI-9).

The misalignment of the eye movement responses shown in Figure 3A are displayed in Figure 3B. On a group level, no clear and consistent positive or negative trend could be observed.

### 3.2 Effect of phase duration

**Figure 4:**
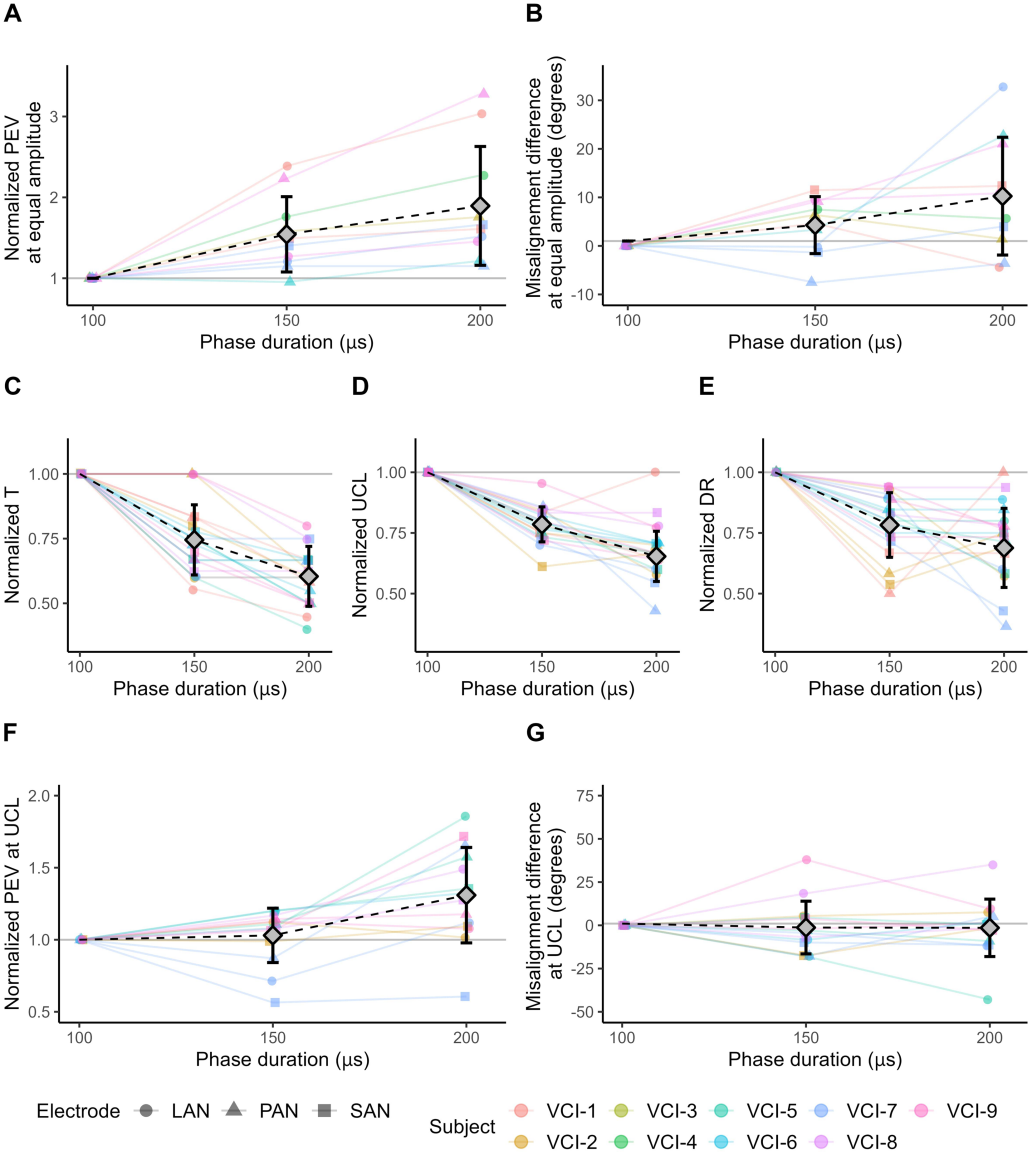
VI response characteristics as a result of 2 s block stimuli with different phase duration values. Responses evoked with a phase duration of 100 μs are used as reference value for normalization. Diamonds represent mean values, error bars indicate one standard deviation. **(A)** The normalized PEV measured in each electrode with three phase duration settings. For each subject/electrode/experiment combination, the amplitude was equal and was chosen at the highest level measured in all three settings. **(B)** Misalignment difference of the responses shown in **(A)**. **(C)** Normalized threshold (T) amplitude levels. **(D)** Normalized amplitude of upper comfortable limits (UCL). **(E)** Normalized dynamic ranges (DR). **(F)** Normalized PEV at the upper comfortable limit (UCL). UCL can differ within subject/electrode series. **(G)** Misalignment difference at the upper comfortable limit (UCL). **(A,B,F,G)** Only electrodes showing eVOR responses (PEV >30 °/s) in all three conditions are displayed.

Figure 4 presents the effects of using different phase durations (100, 150, and 200 μs) on the response characteristics.

All included electrodes (PEV >30°/s) evoked a higher PEV when stimulated with a longer phase duration and equal stimulus amplitude (Figure 4A). Average increase in PEV was 54% (SD 47%) between the phase duration at 100 and 150 μs, and 89% (SD 73%) between 100 and 200 μs.

Additionally, the increase in PEV as a result of the longer phase duration did not depend on the PEV of the response at phase duration of 100 μs. This was illustrated by the difference in PEV between phase duration settings 150 μs versus 100 μs, and 200 μs versus 100 μs. These differences were only weakly associated with the PEV at phase duration of 100 μs (r = −0.17 for phase duration of 150 μs; and −0.35 for phase duration of 200 μs).

The misalignment difference of the responses presented in Figure 4A are shown in Figure 4B. Compared to a phase duration of 100 μs, the mean difference was 4.28° (SD 5.87°) and 10.25° (SD 12.14°) at phase duration of 150 and 200 μs, respectively.

The use of longer phase durations resulted in lower thresholds in all electrodes (Figure 4C). The average decrease in threshold was 25% (SD 14%) between phase duration of 100 nd 150 μs, and 40% (SD 12%) between 100 and 200 μs.

Furthermore, a shorter phase duration resulted in a higher UCL in almost all electrodes (Figure 4D). This involved eight out of eight electrodes limited by perception, and 17 out of 19 electrodes limited by facial nerve stimulation. Compared to a phase duration of 100 μs, the average decrease in UCL was 21% (SD 7%) and 35% (SD 10%) for the phase durations of 150 and 200 μs, respectively.

Next, to the UCL, the dynamic range decreased on average by 22% (SD 13%) from phase duration of 100 to 150 μs, and 31% (SD 16%) from 100 to 200 μs (Figure 4E). However, the decrease in dynamic range was not always consistent with increases in phase duration, as the dynamic range at phase duration of 200 μs was in 10 electrodes equal to, or greater than the dynamic range at 150 μs.

The PEV at UCL generally increased with longer phase durations (Figure 4F). Of the 14 electrodes (with a PEV >30 °/s in at least one of the three phase duration conditions), 13 showed a higher PEV when comparing a phase duration of 200 to 100 μs. On average, the PEV was 3% (SD 19%) higher with phase duration of 150 μs compared to 100 μs, and 31% higher (SD 33%) for 200 μs in comparison to 100 μs.

The misalignment of the eye movement responses shown in Figure 4F are displayed in Figure 4G. On average, misalignment was not affected by the phase duration. It was -1.28° (SD 15.23°) lower with a phase duration of 150 μs compared to 100 μs, and −1.45° (SD 16.60°) lower for 200 μs compared to 100 μs.

### 3.3 Effect of pulse rate

**Figure 5:**
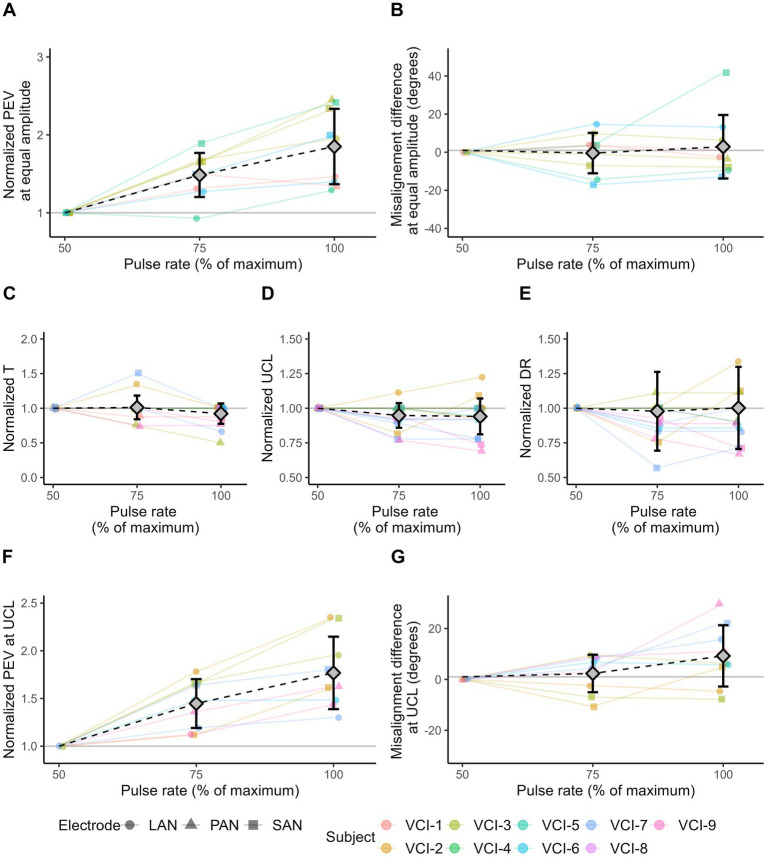
VI response characteristics as a result of 2 s block stimuli with different pulse rates. Responses evoked with a pulse rate at 50% are used as reference values for normalization. Diamonds represent mean values, error bars indicate one standard deviation. **(A)** Normalized PEV measured in each electrode with three pulse rate settings. For each subject/electrode/experiment combination, the amplitude was equal and selected based on the highest level measured in all three settings. **(B)** Misalignment difference of the responses shown in **(A)**. **(C)** Normalized threshold (T) amplitude levels. **(D)** Normalized amplitude of upper comfortable limits (UCL). **(E)** Normalized dynamic ranges (DR). **(F)** Normalized PEV at the upper comfortable limit (UCL). UCL could differ within subject/electrode series. **(G)** Misalignment difference at the upper comfortable limit (UCL). **(A,B,F,G)** Only electrodes showing eVOR responses (PEV >30 °/s) in all three conditions are displayed.

Figure 5 presents the effects of different pulse rates (50, 75, and 100% of the maximum achievable rate) on the response characteristics.

All electrodes evoked a higher PEV at higher pulse rates (Figure 5A). Average increase in PEV was 54% (SD 46%) between pulse rates 50 and 75%, and 89% (SD 73%) between pulse rates 50 and 100%.

Additionally, the increase in PEV as a result of the higher pulse rate, depended on the PEV of the response at 50% pulse rate. This was indicated by the difference in PEV between pulse rate settings 75 versus 50%, and 100% versus 50%. These differences were strongly correlated with the PEV at pulse rate 50% (*r* = 0.77 for pulse rate of 75%; and 0.68 for pulse rate of 100%).

The misalignment of the responses shown in Figure 5A are shown in Figure 5B. Misalignment differences were distributed around zero. The average change in misalignment was -0.45° (SD 10.60°) between pulse rates 50 and 75%, and 2.88° (SD 16.66°) between pulse rates 50 and 100%.

On average, no difference in threshold was found with higher pulse rates (Figure 5C). Differences were distributed around zero (mean at 75% pulse rate was 1%, SD 17%; mean at 100% pulse rate was -8%, SD 15%).

In general, electrodes showed no or minimal differences in UCL with higher pulse rates (Figure 5D). Differences were distributed around zero (mean at 75% pulse rate was -5%, SD 9%; mean at 100% pulse rate was -6%, SD 13%).

In line with the threshold and UCL, the electrodes generally showed no or minimal differences in dynamic range with higher pulse rates (Figure 5E). Differences were distributed around zero (mean 75% rate is -2%, SD 28%; mean 100% rate is 0.2%, SD 31%).

All electrodes had a higher PEV at UCL when evoked with a higher pulse rate compared to the 50% pulse rate (Figure 5F). On average, the PEV was 45% (SD 26%) higher with pulse rate of 75 compared to 50%, and 77% (SD 38%) higher for pulse rate 100% compared to 50%.

The misalignment of the responses from Figure 5F are shown in Figure 5G. On average, the misalignment was 2.33° (SD 7.37°) higher with pulse rate 75% compared to 50%, and 9.24° (SD 12.05°) higher for pulse rate 100% compared to 50%.

### 3.4 Charge comparisons

By doubling the phase duration, and therefore doubling charge, on average 60% of the amplitude was needed to reach activation threshold (Figure 4C). Hence, approximately 20% more charge was needed to reach the threshold with a phase duration of 200 compared to 100 μs (2*normalized T at 200 μs). On the contrary, when doubling the pulse rate, on average the threshold of activation decreased by only 8% (Figure 5C). Consequently, approximately 84% more cumulative charge was needed to reach the threshold (2*normalized T at 100%). UCL demonstrated the same pattern. To reach UCL, on average an increase in 58% cumulative charge was required when doubling phase duration (Figure 4D; 2*normalized UCL at 200 μs), and an increase of 88% when doubling pulse rate (Figure 5D; 2*normalized UCL at 100%).

**Figure 6:**
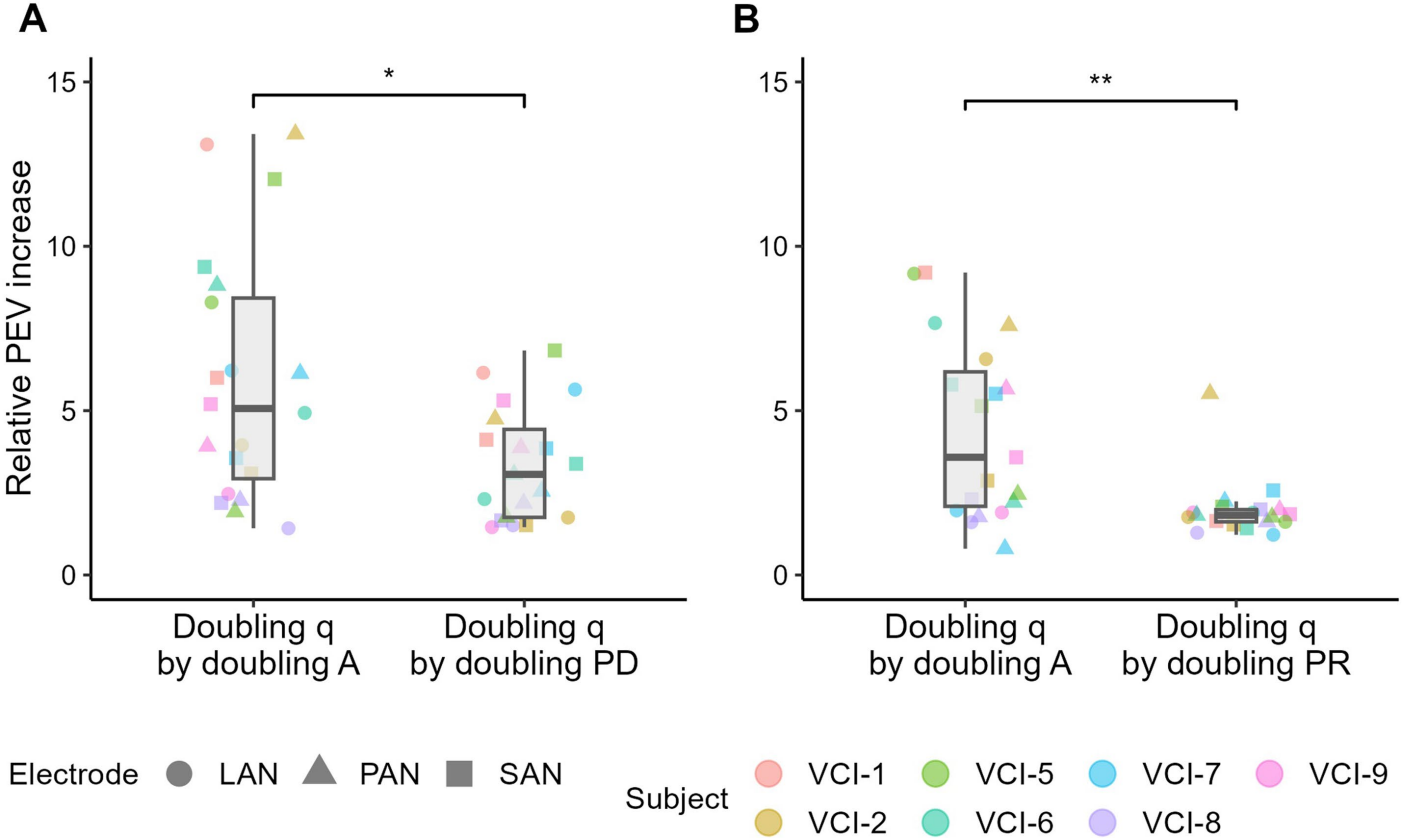
PEV, charge (q) based, comparison between **(A)** the stimulation amplitude (A) and phase duration (PD) and **(B)** between the stimulation amplitude and pulse rate (PR). Comparison is made by doubling the delivered charge by doubling the amplitude, phase duration and stimulation rate. In each figure, the paired samples have the same delivered charge. Relative PEV increase was calculated by dividing the doubled charge PEV by the initial PEV. Boxplots indicate the median and interquartile ranges of the relative PEV increase. Paired two-sided Wilcoxon signed-rank tests were performed, with * indicating *p* < 0.001. Note that the data was obtained in two experiments (amplitude versus phase duration, and amplitude versus pulse rate), which explains the difference between the data in the two “doubling q by doubling A” boxplots.

The mean of the individual relative increase in PEV was a factor 5.92 when doubling the charge by doubling the amplitude, and 3.35 when doubling the phase duration (Figure 6A). In the second experiment, the relative PEV increase was a factor 4.41 when doubling the amplitude, and 1.98 when doubling the rate (Figure 6B). Paired-samples two-sided Wilcoxon signed-rank tests indicated statistical significant differences in both comparisons (with *p <* 0.001).

### 3.5 Effect of stimulus shape

**Figure 7:**
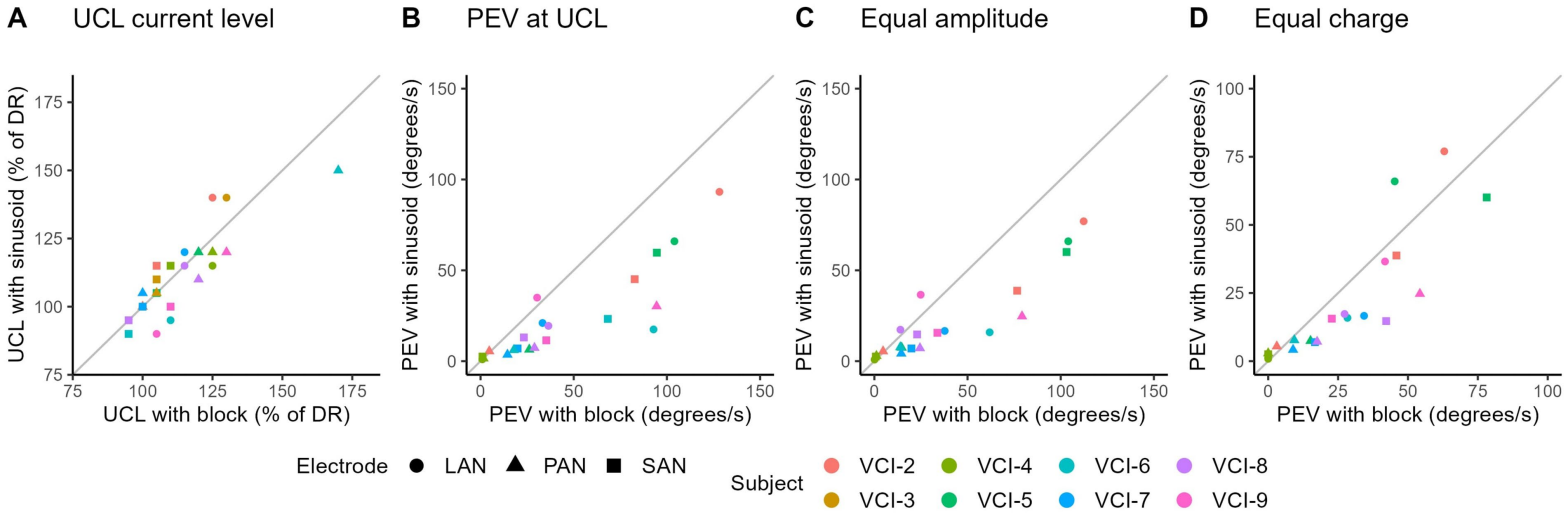
Comparison between block and sinusoidal stimulation on different vestibular implant response characteristics and in different settings. All markers represent the average of two measurements with block, and two measurements with sinusoidal stimulations. **(A)** Comparison of upper comfortable limit (UCL). **(B)** PEV at UCL (note, UCL can differ between block and sinusoidal stimulations). **(C)** PEV comparison of sinusoidal versus block stimulation with equal amplitudes. **(D)** PEV comparison of sinusoidal versus block stimulation with equal charge levels.

Figure 7 shows the comparison between sinusoidal and block stimulation on different vestibular implant response characteristics and with varying settings.

On average, the UCL measured with sinusoids was 8 cu higher than those with block stimulation (SD 8 cu) (Figure 7A). As the points varied around the diagonal, no clear difference was found in UCL between the use of sinusoidal or block shaped stimuli.

The UCL was limited by either patient discomfort or facial nerve stimulation. UCL’s limited by patients discomfort and facial nerve stimulation both showed negligible differences in UCL. However, on average the UCL tended to be higher with sinusoidal stimulation when limited by facial nerve stimulation. On the other hand, when limited by patient discomfort (e.g., pain), the UCL tended to be lower with sinusoidal stimulation. Those observations were derived from the following comparisons. The eight subjects, each with three electrodes measured twice, provided a total of 48 comparisons. Out of 48 comparisons, 29 showed a UCL limited by facial nerve stimulation, of which 15 had a higher UCL with sinusoids (average difference 10.71%, note step size is 10%). Next to the 29, 10 had equal UCL levels, and four had a lower UCL with sinusoids (average 10%, one step). The other 19 UCL levels were limited by patient discomfort, of which 12 had a lower UCL with sinusoidal stimulation (average 15%). Next to the 12, five had equal UCL, and two had a higher UCL with sinusoidal stimulation (average 10%).

Furthermore, stimulating at UCL resulted in a higher PEV with block stimulation in almost all electrodes (Figure 7B). When comparing sinusoidal and block stimulation using equal amplitudes, block stimulation evoked responses with a higher PEV in most electrodes (Figure 7C). This result was generally the same when using equal charge (Figure 7D).

## 4 Discussion

In this study, the basic stimulation parameters of vestibular implant stimulation were investigated, with the aim of optimizing the electrically evoked vestibulo-ocular reflex. The stimulation amplitude showed to be strongly correlated with PEV, but not with the misalignment. Additionally, it was found that the maximum PEV can be increased by increasing the phase duration and pulse rate. Both parameters have little effect on misalignment. However, a longer phase duration decreased the dynamic range. An overview of the observations is provided in Table 2. Furthermore, this study demonstrated that next to the amplitude of stimulation, the stimulus shape has a determinative effect on the PEV.

**Table 2:** Summary of the effects on the response parameters when the amplitude, phase duration and pulse rate are increased.

Parameter	PEV		Misalignment		UCL	T	DR	q needed
	At same A	At UCL	At same A	At UCL				
Amplitude ↑	**↑**		**≈**		NA	NA	NA	NA
Phase duration **↑**	**↑**	**↑**	**↑**	**≈**	**↓**	**↓**	**↓**	**↑**
Pulse rate **↑**	**↑**	**↑**	**≈**	**↑**	**≈**	**≈**	**≈**	**↑**

A, amplitude; UCL, upper comfortable limit; T, threshold; DR, dynamic range; “q needed,” charge needed to reach T and UCL; NA, not applicable.

### 4.1 Pulse amplitude

Stimulation amplitude was strongly correlated with the PEV. This observation is in line with previous literature (7, 15). The strong correlation makes it a suitable parameter to encode vestibular information. However, increasing the amplitude imposes a risk of spread of excitation, potentially activating non-target neural substrates such as the neighboring facial and ampullary nerves. Activation of the neighboring ampullary nerves results in misalignment, potentially disturbing the interpretation of the applied stimulation. However, this effect was not consistently observed in our subjects. The misalignment did not increase with increased stimulus amplitude on group level, but it did occur in a subset of electrodes. This indicates that the spread of excitation generally either remained below the activation threshold of the adjacent ampullary nerves, or that the ratio of activation over the ampullary nerves remained equal with increasing amplitude. The latter is most probable, as a degree of misalignment was present in the majority of measured responses, indicative of adjacent ampullary nerve activation. This occurred even when stimulation levels were low. The anatomical proximity of LAN and SAN did not seem to increase the probability of interference between those two electrodes relative to PAN. Generally, it was observed that the UCL was often limited by the facial nerve activation threshold, supporting the obvious statement that increased amplitude resulted in increased current spread.

### 4.2 Phase duration

In addition to the amplitude, the phase duration is known to be a relevant parameter in vestibular implant stimulation (15, 16). In order to optimize PEV and stay within compliance limits of the previously used devices, relatively long phase durations were used (e.g., 200 μs). These were relatively long compared to phase durations used in cochlear implant stimulation (<50 μs) (31). However, as mentioned before, applying a shorter phase duration can be beneficial as it potentially results in a wider dynamic range, better spatial selectivity, less power consumption and enabling a higher stimulation rate. During all experiments described in this article, the stimulation levels stayed far below compliance limits, allowing it to examine the potential of using shorter phase durations.

Firstly, as expected, it was found that phase duration is positively correlated with PEV. This was already hypothesized, as a longer phase duration is positively correlated results in more delivered charge. For vestibular implant fitting, it is more relevant to look at the maximum achievable PEV, rather than to look at the effect of the phase duration on the PEV itself. The functional maximum response of the vestibular implant, in terms of PEV, is also determined by the UCL with the used settings. The results of this study demonstrated that the UCL decreased with increased phase duration. As a consequence in terms of functionality, the PEV at UCL is more representative. The effect varied per subject/electrode, but showed a general tendency towards a higher PEV at UCL with a phase duration of 150 and 200 μs.

Previous research showed a correlation between phase duration and PEV in specific settings. The PEV remained constant at a baseline amplitude of 30–50% of the dynamic range, while a trend seemed visible at 70% of the dynamic range (16). It should be noted that the PEV at UCL was not considered (as stated above: UCL is related to phase duration). This implies that the influence of phase duration on maximum achievable PEV was not determined, explaining the difference with our observations.

In addition to maximum PEV, the dynamic range is also an important parameter. A negative correlation between phase duration and dynamic range was found. This is in line with previous results (16, 18). A wider dynamic range provides a larger range to code vestibular information. This offers potential benefit in terms of resolution of interpreted head movements.

The dynamic range is a result of the threshold and UCL level. The phase duration showed a negative correlation with both threshold and UCL. The effect on UCL was found to be stronger, resulting in a wider dynamic range when using a shorter phase duration.

The UCL was often limited by facial nerve stimulation and was higher with shorter phase durations. This implies that pulses with a shorter phase duration are more spatially selective, or that the facial nerve activation threshold is charge-dependent. It was shown that doubling the charge by doubling the phase duration, only leads to a 35% decrease in UCL. This indicates that it is not solely a charge-related effect.

It appeared that the phase duration did not affect the degree of misalignment, when compared with equal amplitudes or at UCL. This implies that the phase duration most likely not influences the spread of excitation within the measured range. This contradicts previous findings (15, 18). However, those results were based on simulations and animal experiments, possibly explaining the observed differences.

Altogether, a longer phase duration generally enabled a higher PEV at UCL (with varying effect sizes per subject/electrode). However, increasing the phase duration led to a smaller dynamic range, and required more charge to reach threshold and UCL.

### 4.3 Pulse rate

Pulse rate has previously shown to be of influence in vestibular implant fitting (15, 16). A higher pulse rate can be beneficial in vestibular implant stimulation for several reasons. It enables a higher temporal resolution of the transferred information, and a higher stimulation rate is expected to provide eye responses with a higher PEV.

As expected, pulse rate positively correlated with PEV. This might be expected, as a higher stimulation rate results in more delivered charge, and a healthy vestibular system is based on activation rate modulation (1). As mentioned above, it is more clinically relevant for vestibular implant fitting to look at the maximum achievable PEV. A positive correlation was found between pulse rate and PEV at UCL within the measured range (~170–340 pulses per second). Interestingly, the threshold, UCL, dynamic range and misalignment did not appear to be correlated with the stimulation rate, except for the very weak correlation observed with misalignment. The UCL appeared to be often limited by facial nerve stimulation, making it a threshold measure. The threshold is a threshold measure by definition (activation threshold of eVOR and/or perception) and the dynamic range is directly derived from the threshold and UCL. Misalignment can be considered a threshold of activation of adjacent ampullary nerves. Hence, this makes them all activation threshold measures. The independency between pulse rate and different activation thresholds is in line with the findings of Crétallaz et al. (16), who stated that activation thresholds are not affected by changing stimulation rate. This was explained by the nerve activation model previously described by DiGiovanna et al. (24). Individual nerve fibers show amplitude dependent sensitivity. This means that with the same amplitude, the same pool of fibers is susceptible for the applied stimulation, generally irrespective of the pulse rate. Increasing the pulse rate will generally only result in a faster activation rate of those nerve fibers, while a larger amplitude increases the pool of susceptible fibers (and therefore also increases the number of pulses over the nerve bundle). As nerve activation is also a stochastic process, an increased pulse rate will increase the number of activated fibers. However, it appeared to be a minor contribution relative to an increased pool of susceptible fibers, achieved through an increase in phase duration. Combined with our results, this implies that the thresholds are more determined by the number of activated fibers, rather than the activation rate.

However, PEV at UCL appeared pulse rate dependent. This indicates that for interpretation of electrical vestibular stimulation, both the number of activated fibers and the activation rate are relevant. They both contribute to the overall activation rate over the whole nerve bundle. This is in line with the fact that a healthy vestibular system is based on activation rate modulation of the ampullary nerve bundle.

In summary, a higher pulse rate resulted in a higher PEV at UCL, generally without an effect on the threshold, UCL, dynamic range and misalignment. Besides the potential increased power consumption, this favors using a higher stimulation rate for vestibular implant stimulation.

### 4.4 Comparison based on charge

To identify whether the effects of stimulus amplitude, phase duration and pulse rate have different characteristics or are commonly charge related, a comparison was made between their effectiveness in terms of, threshold level, UCL and PEV. Concerning activation threshold, 20% more charge was needed to reach threshold with a phase duration of 200 compared to 100 μs (doubling in charge). A pulse rate of 50% needed 100% more charge compared to a pulse rate of 100% (doubling in charge). The UCL showed the same pattern. On average, pulse amplitude demonstrated the largest effect on PEV, followed by phase duration, and pulse rate having smallest effect. Conclusively, this indicates that these parameters have different effects, which are not fully explained by the delivered charge.

### 4.5 Sinusoidal versus block stimulation

To further clarify the response mechanism to vestibular implant stimulation, different stimulus shapes were tested. Block and sinusoidal stimulations were compared to determine relative importance of amplitude and speed of change in stimulation. The UCL was generally equivalent for both sinusoidal and block stimulations for the tested electrodes. This was expected as the UCL was mainly limited by facial nerve stimulation, which was amplitude dependent. The PEV with block stimulations at UCL was higher than those evoked with sinusoidal stimulations at UCL. As the UCL stimulation level was equivalent, this indicates that block stimulations evoked higher eye responses than sinusoidal stimulation at equal amplitude levels. That observation is in line with the equal amplitude comparison, which demonstrated exactly this phenomenon. Altogether, this implies that the system is also responsive to the speed of change, and not solely the absolute amplitude. Moreover, this is congruent with previous literature, which showed the frequency dependency of the vestibular implant response (27). However, when comparing stimulus shapes with equivalent charge, the difference was less pronounced. This in turn indicates that the response can also be charge dependent. On the other hand, in this setting the sinusoid had a higher amplitude, which could compensate for the lower speed of change of stimulation.

Concerning vestibular implant fitting, it does not seem to be relevant which stimulus shape is chosen to determine UCL, as the results were equivalent. However, when the PEV measurements are used for fitting, it is important to consider the stimulus shape since block stimuli result in higher PEVs. In the consideration, the importance of the physiological relevance of the stimuli (i.e., does it represent a common head movement), and the need for measuring the maximum potential of the system, should be taken into account.

### 4.6 Limitations

At the current stage of vestibular implant related research, the sample size is an unavoidable limitation. The fact that this study was conducted systematically, included nine subjects, with all three functioning electrodes, is a relatively large sample size for the field. However, a challenge remains for the statistical analysis to find conclusive answers with sample sizes as used in this study. Secondly, parameter interactions could not be completely elucidated as not all needed parameters combinations could be tested in the current setup without exceeding the acceptable subject burden. This also holds for potential factors of influence, such as the type of nerve stimulated (LAN/SAN/PAN), and inter-subject differences. Additionally, the studied parameters should be examined on a broader range, identifying whether the correlations show equivalent patterns outside of the currently tested range. Lastly, torsional eye movement components could not be obtained. These components should be considered in the future, providing better evaluation of eye responses during 3-dimensional head rotations. New experiments should further explore the vestibular implant response in order to optimize vestibular implant stimulation.

## 5 Conclusion

In this study, the basic parameters of vestibular implant stimulation were tested, with the aim of optimizing the response. It was found that the PEV can be increased by increasing the phase duration and pulse rate. Both parameters have little effect on the misalignment. However, a longer phase duration decreased the dynamic range. Furthermore, these results show that next to the amplitude of the stimulation, the stimulus shape has a deterministic effect on the PEV.

## Data Availability

The raw data supporting the conclusions of this article will be made available by the authors, without undue reservation.
